# A petunia ethylene-responsive element binding factor, *PhERF2*, plays an important role in antiviral RNA silencing

**DOI:** 10.1093/jxb/erw155

**Published:** 2016-04-19

**Authors:** Daoyang Sun, Raja Sekhar Nandety, Yanlong Zhang, Michael S. Reid, Lixin Niu, Cai-Zhong Jiang

**Affiliations:** ^1^Department of Landscape Architecture and Arts, Northwest A&F University, Yangling, Shaanxi 712100, China; ^2^Department of Plant Sciences, University of California Davis, Davis, CA 95616, USA; ^3^Department of Plant Pathology, University of California Davis, Davis, CA 95616, USA; ^4^Crops Pathology and Genetic Research Unit, United States Department of Agriculture, Agricultural Research Service, Davis, CA 95616, USA

**Keywords:** Argonaute, *cucumber*, *mosaic*, *virus*, dicer-like enzyme, RNA-dependent RNA polymerase, *tobacco*, *rattle*, *virus*, transcription factor, virus-induced gene silencing.

## Abstract

Virus-induced RNA silencing is involved in plant antiviral defense and requires key enzyme components, including RNA-dependent RNA polymerases (RDRs), Dicer-like RNase III enzymes (DCLs), and Argonaute proteins (AGOs). However, the transcriptional regulation of these critical components is largely unknown. In petunia (*Petunia hybrida*), an ethylene-responsive element binding factor, *PhERF2*, is induced by *Tobacco rattle virus* (TRV) infection. Inclusion of a *PhERF2* fragment in a TRV silencing construct containing reporter fragments of *phytoene desaturase* (*PDS*) or *chalcone synthase* (*CHS*) substantially impaired silencing efficiency of both the *PDS* and *CHS* reporters. Silencing was also impaired in *PhERF2*- RNAi lines, where TRV-*PhPDS* infection did not show the expected silencing phenotype (photobleaching). In contrast, photobleaching in response to infiltration with the TRV-*PhPDS* construct was enhanced in plants overexpressing *PhERF2*. Transcript abundance of the RNA silencing-related genes *RDR2*, *RDR6*, *DCL2*, and *AGO2* was lower in *PhERF2*-silenced plants but higher in *PhERF2*-overexpressing plants. Moreover, *PhERF2*-silenced lines showed higher susceptibility to *Cucumber mosaic virus* (CMV) than wild-type (WT) plants, while plants overexpressing *PhERF2* exhibited increased resistance. Interestingly, growth and development of *PhERF2*-RNAi lines were substantially slower, whereas the overexpressing lines were more vigorous than the controls. Taken together, our results indicate that PhERF2 functions as a positive regulator in antiviral RNA silencing.

## Highlight

*PhERF2*, an ethylene-responsive element binding factor, acts as a positive transcriptional regulator in antiviral RNA silencing and is essential for efficient silencing of genes in plants.

## Introduction

Virus-induced gene silencing (VIGS) is a rapid and effective method for functional characterization of genes in a plant. A recombinant plant virus carrying a host-derived sequence fragment initiates RNA-mediated post-transcriptional gene silencing (PTGS), leading to a transient and specific degradation of the corresponding endogenous mRNA (Kumagai *et al.*, 1995; Dinesh-Kumar *et al.*, 2003; Di Stilio *et al.*, 2010; Tian *et al.*, 2014). A modified *Tobacco rattle virus* (TRV) vector has proved to be an excellent tool for VIGS, due to its wide host range and its ability to infect meristematic cells (Di Stilio *et al.*, 2010). This system is composed of binary transformation plasmids, TRV1 and TRV2, with a region harboring multiple cloning sites in TRV2 (Liu *et al.*, 2002). The TRV vector has been used successfully for gene function analysis in a number of eudicots, including the commonly used model plants *Arabidopsis* (Turnage *et al.*, 2002; Burch-Smith *et al.*, 2006; Wang *et al.*, 2006), *Nicotiana benthamiana* (Jones *et al.*, 2006; Peterson *et al.*, 2013), *N. tabacum* (Lukhovitskaya *et al.*, 2013), tomato (*Solanum lycopersicum*; Liu *et al.*, 2002; Fu *et al.*, 2005; Jiang *et al.*, 2008; Senthil‐Kumar and Mysore, 2011; Fragkostefanakis *et al.*, 2014) and petunia (*Petunia hybrida*; Chen *et al.*, 2004; Spitzer *et al.*, 2007; Broderick and Jones, 2014; Chang *et al.*, 2014). In petunia, silencing of *phytoene desaturase* (*PDS*) or *chalcone synthase* (*CHS*) provides useful phenotypical markers in VIGS studies for functional characterization of genes in leaf and floral tissues, respectively (Chen *et al.*, 2004; Reid *et al.*, 2009; Jiang *et al.*, 2011).

The silencing efficiency of the VIGS system is variable, largely depending on post-inoculation growth temperature and compatibility between the host and the virus. Growth temperature seems to have profound effects on the efficiency of VIGS-based gene silencing. Gene silencing efficiency with a TRV system in tomato is enhanced by low temperature and low humidity (Fu *et al.*, 2006). Low temperature also enhances gene silencing efficiency throughout the life of cotton plants when geminivirus-mediated VIGS is employed (Tuttle *et al.*, 2008). But in contrast, Szittya *et al.* (2003) reported that low temperature inhibits silencing by preventing siRNA generation in *N. benthamiana* protoplasts transfected with *Cymbidium* ringspot virus (CymRSV). The underlying mechanisms of these apparently conflicting findings are not yet well understood. Previously, we tested the effects of silencing *CHS* on a range of purple-flowered petunia cultivars and found significant variations in the silencing phenotypes (Chen *et al.*, 2004; Reid *et al.*, 2009). In studies with silencing *PDS* in tomato we have also observed cultivar-dependent variations in the silencing phenotype (Jiang *et al.*, 2011). Compatibility has limited the range of taxa where TRV-VIGS has successfully been employed. The genetic basis for the variation and limitation is largely unknown.

The antiviral RNA silencing process involves a set of crucial cellular enzymes including RNA-dependent RNA polymerases (RDRs), Dicer-like RNase III enzymes (DCLs), and Argonaute proteins (AGOs). These components contribute to the generation, cleavage of template double-stranded RNA (dsRNA), and interaction with virus-derived small interfering RNA (vsiRNA), respectively. The vsiRNA is incorporated into a RNA-induced silencing complex (RISC) for post-transcriptional suppression of the homologous RNA molecules (Burch-Smith *et al.*, 2006; Gould and Kramer, 2007; Jaubert *et al.*, 2011; Tian *et al.*, 2014). In *Arabidopsis thaliana*, four DCLs, six RDRs, and ten AGOs serve as critical components of the silencing machinery (Donaire *et al.*, 2008; Zhang *et al.*, 2012). DCL1 has a unique function for the production of 21-nucleotide (nt) microRNAs (miRNAs) that is required for coordination of the complex biological functions within the plant (Dunoyer *et al.*, 2005). DCL2, DCL3, and DCL4 catalyse exogenous RNAs with double-stranded features into 22-, 24- and 21-nt siRNAs (Dunoyer *et al.*, 2005; Donaire *et al.*, 2008; Axtell, 2013), respectively. Double deficiency of DCL2 and DCL4 confers more susceptibility to RNA virus infection, such as TRV (Deleris *et al.*, 2006). Among the six RDRs, RDR1, RDR2, and RDR6 participate in the defensive response against distinct positive-strand RNA viruses through the biogenesis of viral secondary siRNAs in Arabidopsis (Donaire *et al.*, 2008; Garcia-Ruiz *et al.*, 2010; Wang *et al.*, 2010, 2011). Current evidence indicates that AGO1 is mainly implicated in the miRNAs silencing pathway (Baumberger and Baulcombe, 2005). Loss-of-function mutation in AGO2 results in an impaired resistance to 2b suppressor-deficient *Cucumber mosaic virus* (CMV) (Wang *et al.*, 2011) and *Potato virus X* (PVX) (Jaubert *et al.*, 2011) in Arabidopsis, and *Tomato bushy stunt virus* (TBSV) (Scholthof *et al.*, 2011) in *N. benthamiana*. Both AGO1 and AGO2 proteins bind to vsiRNAs (Zhang *et al.*, 2006; Takeda *et al.*, 2008; Wang *et al.*, 2011), thereby guiding the downstream silencing process in the virus-infected cells. To date, however, few transcription factors have been reported to be involved in the transcriptional regulation of these critical components.

We have used petunia as a model system for our studies of flower senescence. Transcriptome analysis has identified a cluster of genes that are strongly up-regulated during development and senescence of the flowers, including many transcription factors (Wang *et al.*, 2013). We have successfully employed the TRV-based VIGS vector and a visual reporter such as *CHS* to study the function of some of these genes (Chen *et al.*, 2004; Reid *et al.*, 2009; Jiang *et al.*, 2011; Chang *et al.*, 2014; Yin *et al.*, 2015). In the course of these studies, we have noted lack of *CHS*-silencing phenotype in the case of simultaneous silencing of *CHS* and a *PhERF2* gene encoding an ethylene response transcription factor. Given the importance of ethylene in plant responses to stress, we hypothesized that this observation might indicate an important role for this transcription factor in the antiviral RNA silencing process. The experiments reported here were designed to test this hypothesis.

## Materials and methods

### Plant materials and growth conditions

Petunia (*Petunia* × *hybrida*, ‘Primetime Blue’, or *Petunia* × *hybrida* ‘Mitchell Diploid’) seeds were planted in a 72-well plastic tray filled with sterile UC soil mix and germinated at room temperature with a 16/8h day/night photoperiod. Seedlings at the 4-leaf stage were transferred to small pots for inoculation with *Agrobacterium* bearing TRV constructs, or infected with CMV, then maintained in a growth chamber at 25/20 ^o^C day/night with a same light/dark cycle. To determine the abundance of gene transcripts in petunia plants inoculated with various TRV constructs, RNA was extracted from the inoculated leaves, the uppermost fully-expanded leaves, or from flowers at anthesis. Young leaves from petunia plants (‘Mitchell Diploid’) grown in a greenhouse under 25/20 ^o^C day/night temperature and natural photoperiods were collected and used for stable transformation.

### Isolation and sequence analysis of *PhERF2*

An 802-bp EST sequence was identified among up-regulated genes during petunia flower development from a microarray analysis (Wang *et al.*, 2013). Using this partial sequence, a further BLAST search in the NCBI Genbank (http://blast.ncbi.nlm.nih.gov/) was carried out to identify a full-length sequence encoding an ethyleneresponsive element binding factor 2, annotated as *PhERF2*. Protein homologues of PhERF2 were identified through the BLAST search in the non-redundant GenBank protein databases. A NCBI web server (http://www.ncbi.nlm.nih.gov/Structure/cdd/wrpsb.cgi) was used to determine the conserved domain. Multiple alignments of the ERF2 proteins and phylogenic tree analysis were carried out using the ClustalW program (http://www.genome.jp/tools/clustalw/) and MEGA4 software.

### Abiotic stress and hormone treatments

To examine the effects of abiotic stress and hormone treatments on the *PhERF2* expression profile, 3-week-old seedlings were used. For the salinity and drought treatments, the plants were placed in a vial with distilled water (control), water containing 100mM NaCl, or without water at room temperature (20 ^o^C). For the cold treatment, the seedlings were placed in a cold room at 4 ^o^C. For the ethylene treatment, the plants were placed in a sealed glass chamber and exposed to 10 µl l^–1^ ethylene in air. For treatments with other hormones, plants were treated with solutions containing 50 μM abscisic acid (ABA), 50 μM gibberellic acid (GA_3_), 200 μM salicylic acid (SA), or 200 μM methyl jasmonate (MeJA). In each case, three individual plants were collected at 0, 3, 6, 12 and 24h post-treatment, then frozen in liquid nitrogen and stored at –80 ^o^C.

### Virus-induced gene silencing

The TRV vectors TRV1 and TRV2 have been described previously (Liu *et al.*, 2002). Our experiments were carried out with previously generated TRV-*PhPDS* or TRV-*PhCHS* constructs, in which a 138-bp *PDS* or 194-bp *CHS* fragment was cloned into the *Eco*RI or *Xba*I-*Eco*RI restriction sites in the TRV2 vector, respectively (Chen *et al.*, 2004). To create the TRV-*PhPDS* or TRV-*PhCHS* construct with a fragment of the gene of interest, *Sac*I and *Xho*I were used to digest the pDAH11 vector containing a 291-bp or 339-bp fragment of *PhERF2* or a 246-bp fragment of *PhERF3*, and the resulting product was ligated into the corresponding site in TRV-*PhPDS* or TRV-*PhCHS* (Chen *et al.*, 2004). The recombinant plasmids were transformed into *Agrobacterium tumefaciens* strain GV3101 using electroporation (Reid *et al.*, 2009; Jiang *et al.*, 2011). The *Agrobacterium* strains were cultured in 15ml LB media (40mg l^–1^ kanamycin, 20mg l^–1^ gentamicin, 10mM MES and 20 μM acetosyringone) at 28 ^o^C in a growth chamber for 48h. *Agrobacterium* cultures were centrifuged at 3000 *g* for 20min, and the pelleted cells were then resuspended in infiltration buffer (10mM MgCl_**2**_, 10mM MES and 200 μM acetosyringone) to an OD600 of 4.0. The suspensions were shaken gently at room temperature for 3–5h before inoculation. Prior to inoculation, *Agrobacterium* cultures bearing the TRV1 and TRV2 or TRV2-derivatives were mixed together in equal volumes to a final OD600 of 2.0. A 1-ml disposal syringe was used to infiltrate the *Agrobacterium* mixture into the leaves of petunia seedlings (Reid *et al.*, 2009; Jiang *et al.*, 2011).

### Semi-quantitative and real-time quantitative RT-PCR

Total RNA was extracted from the leaves and flowers of petunia plants using TRIzol reagent (Invitrogen, Carlsbad, CA, USA), and purified with RNase-free DNase I (Promega, Madison, WI, USA), according to the manufacturer’s protocol. First-strand cDNA was synthesized from 2–5 μg total RNA with M-MLV reverse transcriptase (Invitrogen, Carlsbad, CA, USA). Real-time quantitative RT-PCR was carried out using the SYBR Green PCR Master Mix (2X) in an ABI7300 instrument (Applied Biosystem, Foster City, CA, USA) as previously described (Liang *et al.*, 2014). 26S ribosomal RNA served as an internal control for normalization of cDNA (Chen *et al.*, 2004; Reid *et al.*, 2009). PCR primers to sequences beyond the region of the inserted fragment for silencing were used for determination of transcript abundance of targeted genes. To confirm accumulation of TRV2 by semi-quantitative RT-PCR, two primer pairs were produced (see Supplementary Table S1 at *JXB* online), of which one (TRV2-1) covered the multiple cloning sites (MCS) in TRV2, so that the size of resulting product varied depending on the inserts in the site, whereas the other (TRV2-2), targeted the region upstream of the MCS and generated bands of uniform size (Supplementary Table S1) (Reid *et al.*, 2009).

### Northern blot assay

Total RNA was isolated from the newly expanded leaves and fully open flowers of inoculated petunia plants, and low-molecular-weight RNA was precipitated using a solution of polyethylene glycol (PEG8000) and NaCl (Wang *et al.*, 2004b). Blot hybridization was carried out as previously described (Donaire *et al.*, 2008). The fragments of *PDS* (138 bps) or *CHS* (194 bps) corresponding to the silencing region were labeled with [γ-^32^P] ATP to generate DNA probes for assessing abundance of *PDS*- or *CHS*-derived siRNAs.

### Measurement of ethylene production

Leaves of petunia seedlings inoculated with TRV empty vector were harvested at different time intervals and sealed in a 50-ml plastic tube at 25 ^o^C for 4h. A 3-ml gas sample was taken from the tube using a gas-tight syringe, and injected into a gas chromatograph (GC-8A; Shimadzu, Kyoto, Japan) for measurement of ethylene concentration, as previously described (Chang *et al.*, 2014; Yin *et al.*, 2015).

### Overexpression and RNAi silencing constructs

A 1137-bp DNA fragment containing the ORF region of the *PhERF2* was PCR-amplified using the primer pair oxPhERF2F1 and oxPhERF2R1 (see Supplementary Table S1). The amplified product was cloned into the pDAH11 vector and then transferred to a pGSA1403 vector in the forward orientation to produce the 35S::*PhERF2* construct (Chang *et al.*, 2014; Yin *et al.*, 2015). To generate the RNAi construct, primers rnaiPhERF2F1 and rnaiPhERF2R1 (Supplementary Table S1), carrying a *Spe*I-*Asc*I and a *Bam*HI-*Swa*I adaptor, respectively, were designed to amplify a 339-bp fragment. The amplified PCR products were cloned into the pDAH11 vector. The sense and antisense fragments were released from digestion of the pDAH11 plasmid and cloned into the pGSA1285 binary vector. The constructs were sequenced to confirm their fidelity as previously described (Liang *et al.*, 2014).

### Stable transformation

The generated constructs were introduced into *Agrobacterium tumefaciens* strain LBA4404. The transformed bacteria were selected by incubation on an LB plate containing 25mg l^–1^ chloramphenicol at 28 ^o^C for 72h. One colony was selected and cultured in YEP medium (10g l^–1^ yeast extract, 10g l^–1^ peptone and 5g l^–1^ NaCl) with appropriate antibiotics for 48h, and adjusted to an OD600 of 0.3 using sterile LB media without antibiotics. Leaves of ‘Mitchell Diploid’ petunias were inoculated with *Agrobacterium* and regenerated as previously described (Wang *et al.*, 2013; Liang *et al.*, 2014). Putative transgenic petunia plants were grown to flowering. The seeds were harvested and germinated in MS plates containing 100mg l^–1^ kanamycin for selection (Estrada-Melo *et al.*, 2015; Yin *et al.*, 2015).

### Inoculation assay with CMV

*Cucumber mosaic virus* (CMV) inoculum was obtained from leaves of Oriental hybrid lily (*Lilium*) cultivar ‘Siberia’. Infectious sap was prepared by extracting CMV-infected leaves in 100mM phosphate buffer, pH 7.0 (1:4 w/v). The fully expanded leaves of 4-week-old healthy petunia seedlings were mechanically inoculated with virus preparations as previously described (Hull, 2009). To ensure an efficient infection, the inoculation process was carried out once more after 24h. Subsequently, inoculated plants were maintained in the growth chamber at 22 ^o^C for evaluation of symptoms. Transcript levels for the CMV coat protein were examined by real-time quantitative RT-PCR (Liang *et al.*, 2014; Estrada-Melo *et al.*, 2015).

## Results

### Identification of *PhERF2*

An 802-bp EST corresponding to a putative ethylene-responsive element binding factor 2 (*ERF2*) gene was identified among genes up-regulated during flower development in petunia from a microarray analysis (Wang *et al.*, 2013). A further BLAST search in the NCBI Genbank revealed a full-length mRNA sequence (accession number HQ259596, NCBI) annotated as *PhERF2* (see Supplementary Fig. S1). Analysis of the deduced polypeptide encoded by *PhERF2* using the BLAST search against non-redundant GenBank databases in the NCBI revealed homologous proteins from diverse plant species. Amino acid alignment and phylogenetic analysis showed that PhERF2 is relatively close to PhERF3 in petunia and to other proteins, such as *Solanum lycopersicum* SlJERF1 (NP_001234513), *S. tuberosum* StRAP2.12 (XP_006342909), *Prunus salicina* PsERF2a (ACM49847), *Nelumbo nucifera* NnRAP2.12 (XP_010273830), *Malus domestica* MdRAP2.12 (NP_001280975), *Nicotiana tabacum* NtCEF1 (Lee *et al.*, 2005), *Capsicum annuum* CaPF1 (Yi *et al.*, 2004) and *Arabidopsis thaliana* AtRAP2.12 (Zhao *et al.*, 2012; Ding *et al.*, 2013) (Supplementary Fig. S2).

### Biotic, abiotic stresses and hormone treatments induce *PhERF2* expression

**Fig. 1.**
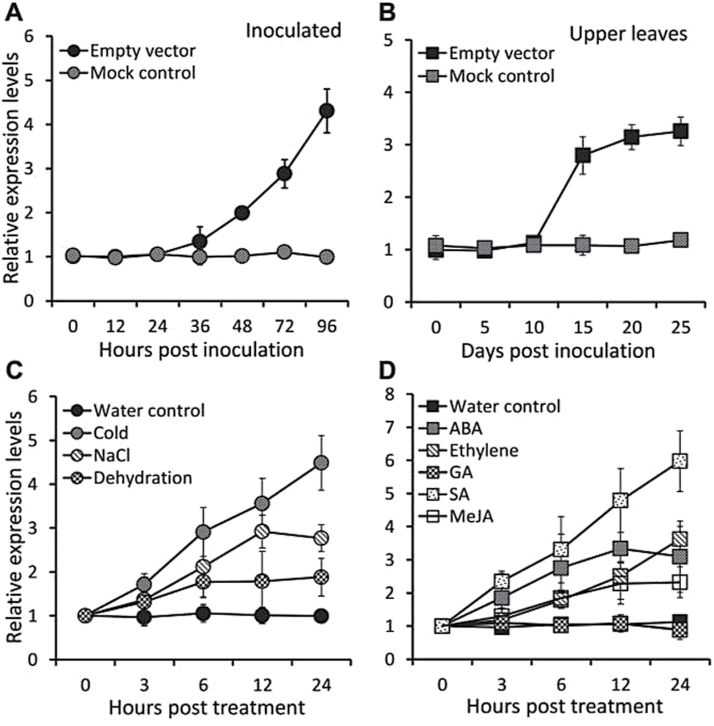
Induction of *PhERF2* expression in petunia leaves infected by TRV, or treated with abiotic stresses or plant hormones. Quantitative RT-PCR analysis of *PhERF2* transcript levels in (A) inoculated and (B) systemically infected (uppermost) leaves at different time points, using 3-week-old WT seedlings infiltrated with *Agrobacterium* bearing no TRV construct (mock control) or TRV empty vector. Quantitative RT-PCR analysis of *PhERF2* transcript abundance in response to abiotic stresses (C) or plant growth regulators (D). Three-week-old WT seedlings were placed in vials with water (control), or treated with a continuous 10 μl l^–1^ ethylene, or with solutions containing either 100mM NaCl, 50 μM ABA, 50 μM GA_3_, 200 μM SA, or 200 μM MeJA, or without water (dehydration) at room temperature, or with water at 4 ^o^C. Transcript abundances were standardized to *26S rRNA*. Error bars represent SE of the means from three biological replicates.

To investigate whether transcript levels of *PhERF2* are induced by TRV infection, wild-type (WT) leaves of petunia plants at the 4-leaf stage were inoculated with *Agrobacterium* bearing an empty TRV vector. *PhERF2* transcripts increased significantly in inoculated leaves by 36h post-inoculation (hpi) (Fig. 1A). By comparison, infection with *Agrobacterium* without the TRV plasmid (mock control) did not affect *PhERF2* transcript levels (Fig. 1A). In systemically infected upper leaves, *PhERF2* transcript abundance increased dramatically between 10 and 15 d post-inoculation (dpi), and remained high thereafter and until 25 dpi (Fig. 1B). As plant hormones and stresses play important roles in the antiviral RNA silencing processes, we examined the expression pattern of *PhERF2* in WT leaves in response to different plant growth regulator and abiotic stress treatments. The expression of *PhERF2* increased following exposure to low temperature, salt stress, and dehydration (Fig. 1C). Transcript levels of *PhERF2* also increased following treatments with SA, ABA, ethylene, and MeJA but not GA_3_ (Fig. 1D).

### Simultaneous silencing of *PhERF2* and *PDS* or *CHS* impairs VIGS efficiency

**Fig. 2.**
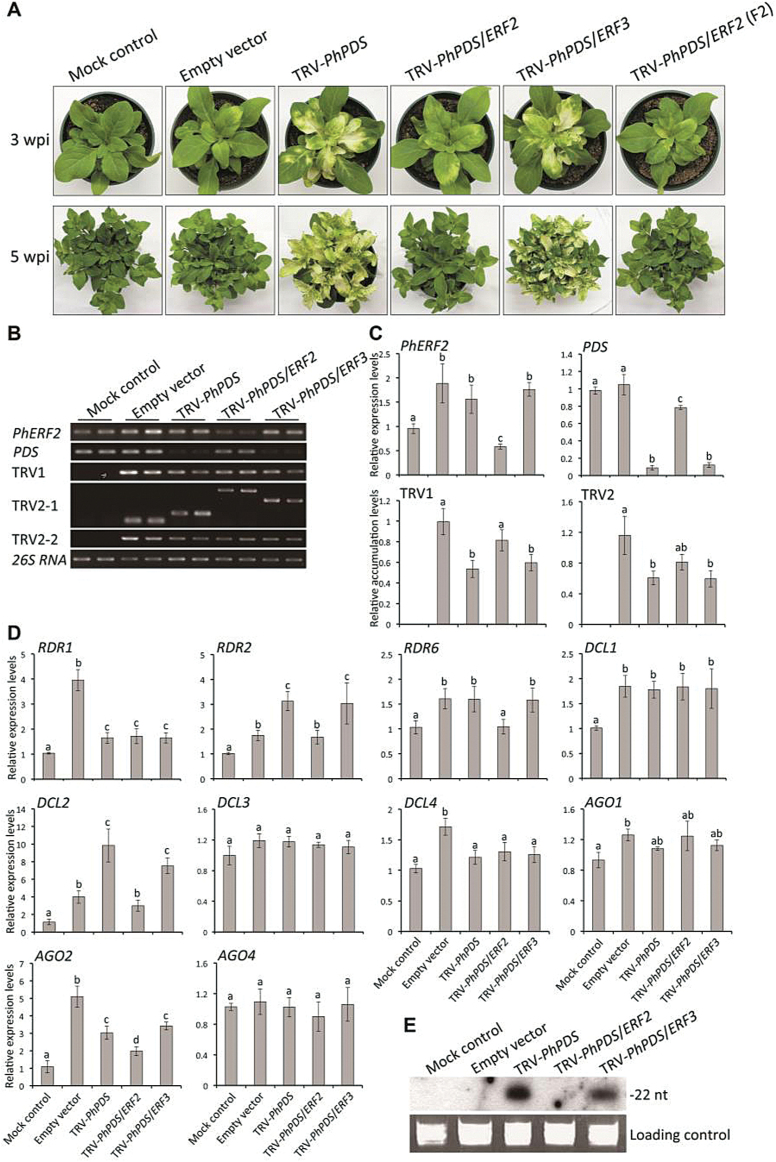
Failed development of photobleaching phenotype in the leaves of VIGS-silenced *PhERF2* plants using *PDS* as a visual reporter. (A) Representative phenotypes of WT plants 3 and 5 weeks post-inoculation (wpi) with non-transformed *Agrobacterium* (mock control), or *Agrobacterium* bearing a TRV empty vector, TRV-*PhPDS*, TRV-*PhPDS*/*ERF2*, TRV-*PhPDS*/*ERF3*, and TRV-*PhPDS*/*ERF2* (fragment 2, F2) constructs. (B), (C) Semi-quantitative and quantitative RT-PCR analysis of transcript abundances for *PhERF2*, *PDS*, TRV RNA1 (TRV1), and TRV RNA2 (TRV2-1, -2) in uppermost younger leaves of plants 3 wpi. Primers for TRV2-1 were designed from the region outside the multiple cloning site (MCS) in the vector and produced variable sizes of products depending on the presence of the *PhPDS*, *PhPDS*/*ERF2*, and *PhPDS*/*ERF3* inserts in the TRV2 vector. Primers for TRV2-2 were used to produce the same sizes of products from the TRV2 vector. Relative accumulation levels were normalized to *26S rRNA*. Error bars represent SE of the means from three biological replicates. Different letters indicate statistical significance as calculated by Duncan’s multiple range test at *P*<0.05. (D) Quantitative RT-PCR analysis of transcript abundances for RNA silencing-related genes, including *RDR1*, *RDR2*, *RDR6*, *DCL1*, *DCL2*, *DCL3*, *DCL4*, *AGO1*, *AGO2*, and *AGO4*, in uppermost younger leaves of plants at 3 wpi. Abundance of *26S rRNA* was used as an internal control. Error bars represent SE of the means from three biological replicates. Different letters denote statistical significance using Duncan’s multiple range test at *P*<0.05. (E) Northern-blot analysis of *PDS* insert-derived siRNA levels in topmost younger leaves of plants at 3 wpi. ^32^P-labeled oligonucleotide probes corresponding to the *PDS* insert sequence were used for detection. Ethidium bromide-stained *5S rRNA* served as a loading control.

**Fig. 3.**
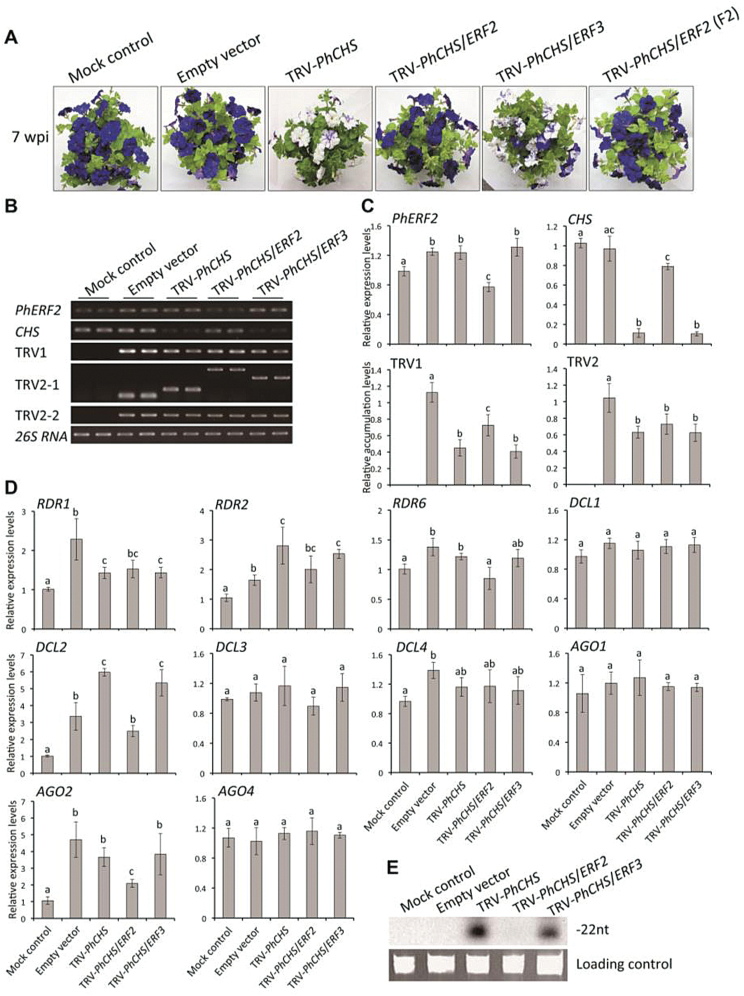
Failed development of the white-corollas phenotype in the flowers of VIGS-silenced *PhERF2* plants using *CHS* as a visual reporter. (A) Representative phenotypes of WT plants 7 weeks post-inoculation (wpi) with non-transformed *Agrobacterium* (mock control), or *Agrobacterium* bearing the TRV empty vector, TRV-*PhCHS*, TRV-*PhCHS*/*ERF2*, TRV-*PhCHS*/*ERF3*, and TRV-*PhCHS*/*ERF2* (fragment 2, F2) constructs. (B), (C) Semi-quantitative and quantitative RT-PCR analysis of transcript abundances for *CHS*, *PhERF2*, TRV RNA1 (TRV1), and TRV RNA2 (TRV2-1, -2) in the corollas. Flowers were harvested at anthesis 7 wpi. Primers for TRV2-1 were designed from the region outside the multiple cloning site (MCS) in the vector and produced variable sizes of products depending on the presence of the *PhCHS*, *PhCHS*/*ERF2*, and *PhCHS*/*ERF3* inserts in the TRV2 vector. Primers for TRV2-2 were used for quantitative RT-PCR to produce the same sizes of products from the TRV2 vector. Abundance of *26S rRNA* was used as an internal control. SE of the means from three biological replicates are indicated by the error bars. Different letters denote statistical significance using Duncan’s multiple range test at *P*<0.05. (D) Quantitative RT-PCR analysis of transcript abundances for RNA silencing-related genes, including *RDR1*, *RDR2*, *RDR6*, *DCL1*, *DCL2*, *DCL3*, *DCL4*, *AGO1*, *AGO2*, and *AGO4*, in the corollas. Flowers were harvested at anthesis 7 wpi. Abundance of *26S rRNA* was used as an internal control. Error bars represent the SE of the means from three biological replicates. Different letters denote statistical significance using Duncan’s multiple range test at *P*<0.05. (E) Northern-blot analysis of *CHS* insert-derived siRNA levels in the corollas, harvested at anthesis, of plants at 7 wpi. ^32^P-labeled oligonucleotide probes corresponding to *CHS* insert sequence were used for detection. Ethidium bromide-stained *5S rRNA* was served as a loading control.

VIGS has proved to be a fast and efficient method to silence genes in petunia. Therefore, to study the function of *PhERF2,* we employed a TRV-based VIGS system to silence *PhERF2* in the leaves and corollas, using *PD*S and *CHS* as visual reporters. The mock-treated and empty vector-inoculated plants showed the WT phenotype with green leaves and purple flowers (Figs 2A and 3A). In plants infected with the TRV-*PhPDS* construct, upper leaves showed a clear *PDS*-silenced photobleaching phenotype by 3 weeks post-infiltration (wpi) (Fig. 2A). The flowers on the plants infected with the TRV-*PhCHS* construct were largely white, corresponding to the silenced phenotype of *CHS* (Fig. 3A).

The plants infected with the TRV-*PhPDS*/*ERF2* or TRV-*PhCHS*/*ERF2* construct, bearing a 291-bp sequence from the 5′ end of the *PhERF2* cDNA, failed to show the normal foliar (*PDS*-photobleaching leaves) and floral (*CHS*-white flowers) silencing phenotypes (Figs 2A and 3A). These results seemed to be specific to *PhERF2*, since infection with a TRV-*PhPDS*/*ERF3* or TRV-*PhCHS*/*ERF3* construct bearing a 246-bp fragment of *PhERF3*, a paralog of *PhERF2* (see Supplementary Fig. S2B), produced the normal silencing phenotypes in leaves and flowers (Figs 2A and 3A). Moreover, infection with a TRV-*PhPDS*/*ERF2* or TRV-*PhCHS*/*ERF2* vector bearing a different 339-bp fragment (Fragment 2) from the 3′ region of the *PhERF2* cDNA also failed to elicit the silencing phenotypes (Figs 2A and 3A).

### VIGS silencing of *PhERF2* does not suppress TRV movement or replication

To understand why upper leaves of plants infected with TRV-*PhPDS*/*ERF2* displayed none of the photobleaching seen in those from plants infected with TRV-*PhPDS* and TRV-*PhPDS*/*ERF3* (Fig. 2A), we performed semi-quantitative and real-time quantitative RT-PCR to analyse transcript abundances of genes including *PhERF2*, *PDS*, *CHS*, TRV RNA1, and RNA2. The results revealed a 50% reduction of *PhERF2* transcript levels in leaves from TRV-*PhPDS*/*ERF2*-infected plants, and 2-fold increases in transcripts in leaves from TRV-*PhPDS*- and TRV-*PhPDS*/*ERF3*-infected plants, compared to the mock control (Fig. 2B, C). *PDS* transcript abundance negatively correlated with the photobleaching in the upper leaves (Fig. 2C). Abundance of *PDS* transcript in the TRV-*PhPDS*- and TRV-*PhPDS*/*ERF3*-infected plants was reduced by over 90% whereas *PDS* abundance was only 20% less than the controls in the TRV-*PhPDS*/*ERF2*-infected plants (Fig. 2C). Accumulation of TRV RNA1 (TRV1) and RNA2 (TRV2) in the leaves of plants agro-infiltrated with an empty vector or TRV-*PhPDS*/*ERF2* constructs was significantly higher than in those of TRV-*PhPDS*- and TRV-*PhPDS*/*ERF3*-infected plants (Fig. 2B, C). Similar results were obtained when petunia plants were infected with the TRV-*PhCHS*/*ERF2* construct using a flower-specific visual reporter (*CHS*) (Fig. 3A–C).

### VIGS Silencing of *PhERF2* affects expression of RNA silencing-related genes

To study the role of *PhERF2* in the RNA silencing process, we determined transcript abundances of a number of putative RNA silencing-related genes. Infection with TRV empty vector or silencing constructs resulted in changes in expression of *RDR*s (*RDR1*, *2*, *6*), *DCL*s (*DCL1*–*4*) and *AGO*s (*AGO1*, *2*, *4*) (Fig. 2D). Almost all the selected genes were up-regulated by infection with the TRV empty vector compared to the mock control (Fig. 2D). Up-regulation was considerably higher for *RDR2*, *DCL2*, and *AGO2* in leaves of plants showing the *PDS*-silenced photobleaching phenotype (TRV-*PhPDS* and TRV-*PhPDS*/*ERF3*) (Fig. 2D). However, this increase was not observed in leaves from TRV-*PhPDS*/*ERF2*-infected plants, in which *RDR6* expression levels were also significantly lower than in photobleached leaves (Fig. 2D).

Using the *PDS* silencing insert as a labeled probe for hybridization, we conducted northern blot analysis to visualize the abundance of TRV-*PhPDS*-derived siRNAs. Leaves from TRV-*PhPDS*- and TRV-*PhPDS*/*ERF3*-infected plants contained a 22-nt RNA fragment that bound to the labeled probe (Fig. 2E). But there was no detectable signal in leaves from TRV-*PhPDS*/*ERF2*-infected plants (Fig. 2E). Similar patterns from the RNA silencing-related gene expression and northern blot analyses were obtained using corollas of petunia plants inoculated with TRV-*PhCHS*-based constructs (Fig. 3D, E).

### *PhERF2*-RNAi silencing impairs VIGS efficiency

**Fig. 4.**
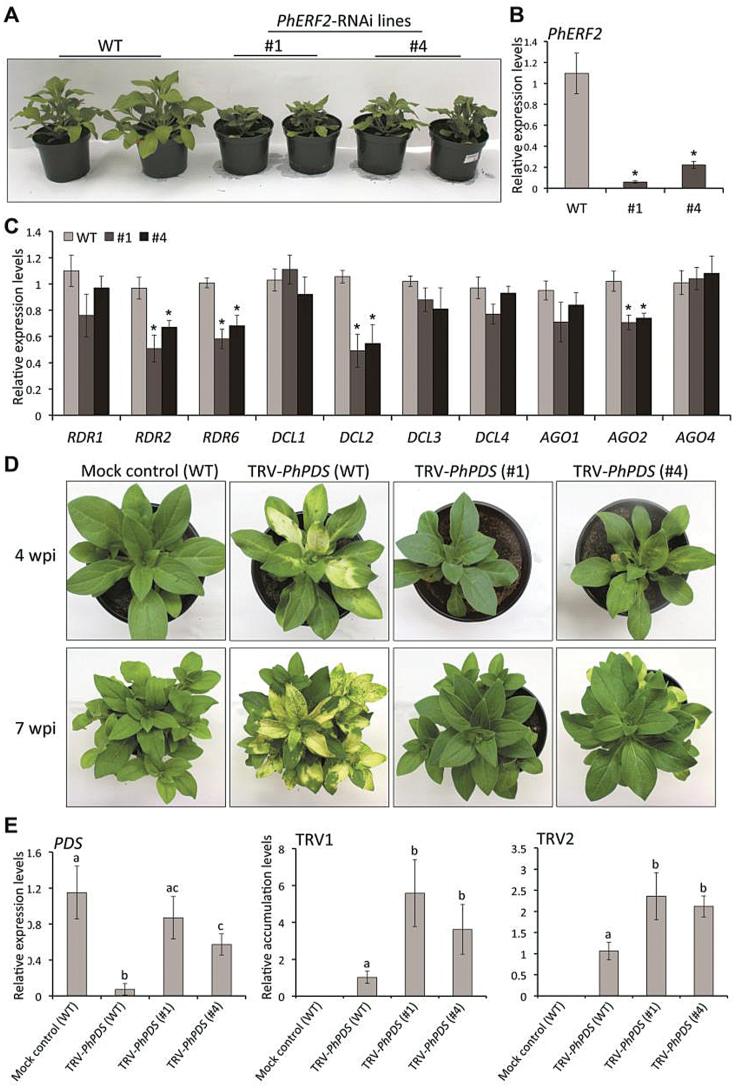
Impairment of photobleaching phenotype in *PhERF2*- RNAi lines inoculated with *Agrobacterium* bearing TRV-*PhPDS*. (A) Representative growth phenotypes of WT and *PhERF2*-RNAi lines (#1 and #4) 40 d post-germination. Note that *PhERF2*-RNAi lines showed delayed growth. (B), (C) Quantitative RT-PCR analysis of transcript abundances for *PhERF2, RDR1*, *RDR2*, *RDR6*, *DCL1*, *DCL2*, *DCL3*, *DCL4*, *AGO1*, *AGO2*, and *AGO4* in the leaves of WT and *PhERF2*-RNAi lines (#1 and #4). Uppermost leaves of 4-week-old plants were used. (D) Representative phenotypes of WT and *PhERF2*-RNAi lines (#1 and #4) inoculated with *Agrobacterium* bearing no TRV vector (mock control) or a TRV-*PhPDS* construct at 4 and 7 weeks post-inoculation. (E) Quantitative RT-PCR analysis of transcript abundances for *PDS*, TRV RNA1 (TRV1), and TRV RNA2 (TRV2) in uppermost leaves of WT and *PhERF2*-RNAi lines (#1 and #4) inoculated with *Agrobacterium* bearing no TRV vector (mock control) or a TRV-*PhPDS* construct. Transcript abundances were standardized to *26S rRNA*. Error bars represent the SE of the means from three biological replicates (B, C, E). Asterisks (B, C) or different letters (E) denote statistical significance using Duncan’s multiple range test at *P*<0.05.

To further study the function of *PhERF2*, we generated *PhERF2*-RNAi lines in petunia. Growth and development of *PhERF2*-RNAi lines was substantially slower than WT plants (Fig. 4A). The *PhERF2*-RNAi lines (#1 and #4) showed substantial reduction in *PhERF2* transcript abundance (Fig. 4B). The transcript abundance of RNA silencing-related genes, including *RDR2*, *RDR6*, *DCL2*, and *AGO2*, was also significantly lower in these RNAi-silencing lines (Fig. 4C). When these lines were infected with the TRV-*PhPDS* silencing construct, they showed no, or much reduced, photobleaching (Fig. 4D), and a concomitant high abundance of *PDS*, TRV RNA1, and RNA2 transcripts in comparison to the photobleached leaves of infected WT plants (Fig. 4E).

### Overexpression of *PhERF2* enhances silencing

**Fig. 5.**
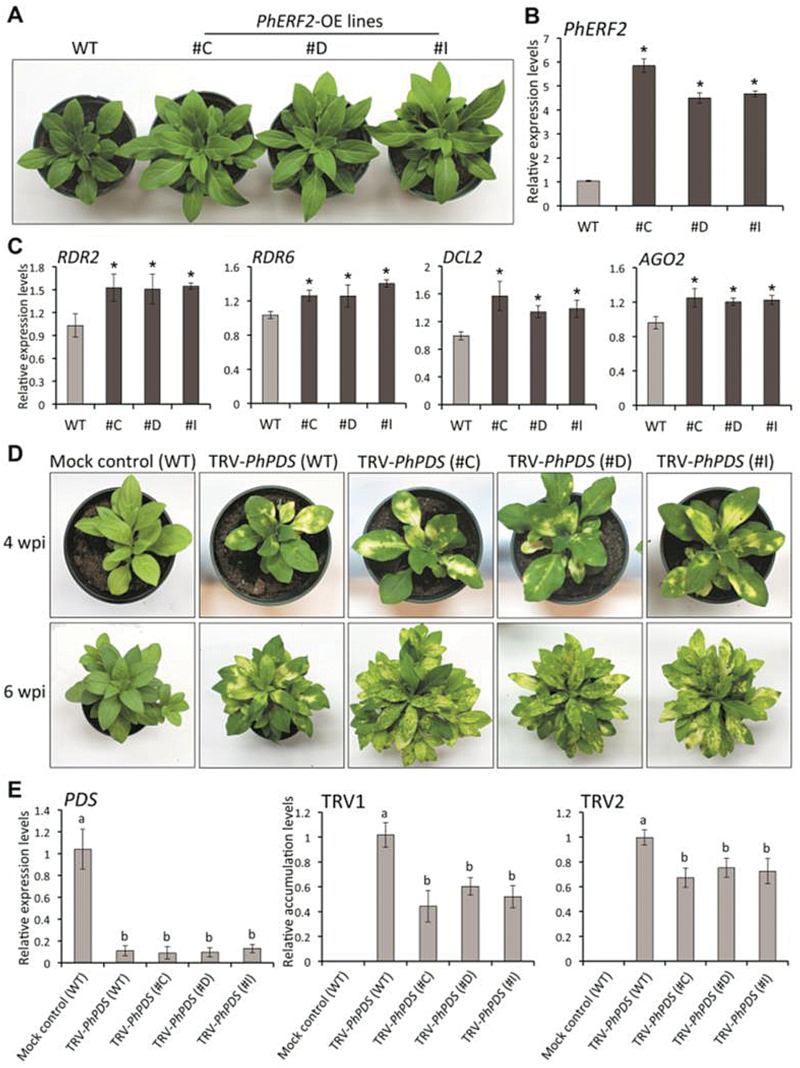
Photobleaching phenotypes of *PhERF2*-overexpressing lines inoculated with *Agrobacterium* bearing a TRV-*PhPDS* construct. (A) Representative growth phenotypes of WT and *PhERF2*-overexpressing (OE) lines (#C, #D and #I) 30 d post-germination. Note that overexpression of *PhERF2* enhanced plant growth. (B), (C) Quantitative RT-PCR analysis of transcript abundances for *PhERF2, RDR2*, *RDR6*, *DCL2*, and *AGO2* in the leaves of WT and *PhERF2*-OE plants (#C, #D and #I). Samples were harvested from uppermost leaves of 4-week-old plants. (D) Representative phenotypes of WT and *PhERF2*-OE lines (#C, #D and #I) inoculated with *Agrobacterium* bearing no TRV vector (mock control) or a TRV-*PhPDS* construct at 4 and 6 weeks post-inoculation (wpi). (E) Quantitative RT-PCR analysis of transcript abundances for *PDS* and TRV RNA1 (TRV1), and TRV RNA2 (TRV2) in uppermost leaves of WT and *PhERF2*-OE lines (#C, #D and #I) inoculated with *Agrobacterium* bearing no TRV vector (mock control) or a TRV-*PhPDS* construct at 4 wpi. Abundance of *26S rRNA* was used as an internal control. Error bars represent the SE of the means from three biological replicates (B, C, E). Asterisks (B, C) or different letters (E) denote statistical significance using Duncan’s multiple range test at *P*<0.05.

To further investigate the role of *PhERF2* in the antiviral RNA silencing process, we also generated *PhERF2* overexpression lines in petunia. Transgenic plants overexpressing *PhERF2* under the control of the *Cauliflower mosaic virus* (CaMV) 35S promoter grew faster (Fig. 5A), and had up to six times the *PhERF2* transcript abundance found in WT plants (Fig. 5B). Transcripts of the RNA silencing-related genes including *RDR2*, *RDR6*, *DCL2*, and *AGO2* were significantly higher in the overexpressing lines than in the WT controls (Fig. 5C). When infected with the TRV-*PhPDS* silencing construct, the *PhERF2*-overexpressing lines showed similar silencing phenotypes and reduction in *PDS* transcripts to those seen in TRV-*PhPDS*-inoculated WT plants (Fig. 5D, E). The accumulation of TRV RNA1 and RNA2 was lower in the overexpressing lines than in the WT when inoculated with TRV-*PhPDS* construct (Fig. 5E).

### *PhERF2* affects disease susceptibility

**Fig. 6.**
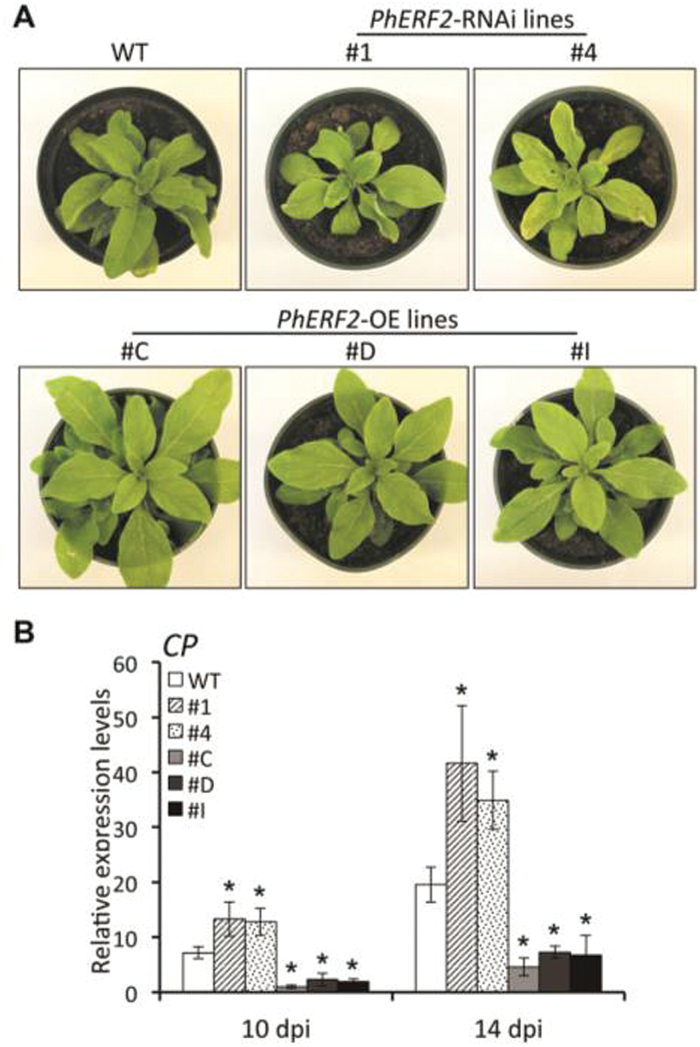
Involvement of PhERF2 in the defense of petunia plants against CMV. (A) Disease symptoms of WT, *PhERF2*-RNAi (#1 and #4), and *PhERF2*-overexpressing (OE) (#C, #D and #I) lines inoculated with CMV at 2 weeks post-inoculation; 4-week-old seedlings were used for inoculation. (B) Quantitative RT-PCR analysis of CMV coat protein (*CP*) transcript abundances in systemically infected (uppermost) leaves of WT, *PhERF2*-RNAi (#1 and #4), and *PhERF2*-OE (#C, #D and #I) lines at 10 and 14 d post-inoculation. Transcript abundances were standardized to *26S rRNA*. Error bars represent the SE of the means from three biological replicates. Asterisks denote statistical significance using Duncan’s multiple range test at *P*<0.05.

Given that *PhERF2* is an important factor in antiviral RNA silencing against TRV, the role of *PhERF2* in response to *Cucumber mosaic virus* (CMV) was then examined. *PhERF2*- RNAi lines inoculated with CMV showed more distortion and yellow mottling of systemically infected leaves than WT plants (Fig. 6A). In contrast, infected *PhERF2*-overexpressing lines had much milder symptoms and continued growth (Fig. 6A). These phenotypes were correlated with measured accumulations of CMV coat protein (*CP*) transcripts, which were significantly higher in *PhERF2*-RNAi lines and dramatically lower in *PhERF2*-overexpressing lines than in WT plants (Fig. 6B).

## Discussion

The data presented here support our hypothesis that the ethylene-responsive element binding factor PhERF2 plays an important role in antiviral RNA silencing (see Supplementary Fig. S3). ERFs belong to the plant-specific AP2/ERF transcription factor superfamily, whose members share a highly conserved AP2/ERF DNA binding domain with a length of 57–66 amino acids (Okamuro *et al.*, 1997). In Arabidopsis, there are a total of 122 putative ERF genes (Nakano *et al.*, 2006). They appear to bind to a *cis*-acting GCC box with a core AGCCGCC motif (Hao *et al.*, 1998) that is commonly found in the promoter region of defense-related genes downstream from the ethylene signaling pathway (Ohme-Takagi and Shinshi, 1995).

Ethylene is the key regulator in the senescence of petunia flowers, and we have been dissecting the control of the process by silencing structural and regulatory genes, including *ERF* genes, in the ethylene response cascade (Wang *et al.*, 2013; Chang *et al.*, 2014; Yin *et al.*, 2015). When we silenced *PhERF2*, using a TRV vector containing a fragment of *PhERF2* and either a foliar or floral reporter gene (*PDS* or *CHS*, respectively), we noted a dramatic reduction in VIGS efficiency, as demonstrated by very little photobleaching in the leaves or white corollas on the normally purple flowers (Figs 2A and 3A). *PhERF2* transcripts were abundant in control plants infected with the empty TRV vector. These findings led to the hypothesis that *PhERF2* might play an important role in the transcriptional regulation of the silencing response system.

Antiviral RNA silencing involves up-regulation of a number of genes, including *RDR*s, *DCL*s, and *AGO*s, that are required for the generation and cleavage of double-stranded RNA (dsRNA) and its processing into virus-derived small interfering RNAs (vsiRNA). In Arabidopsis, four DCLs, six RDRs and ten AGOs serve as critical components of the silencing machinery (Donaire *et al.*, 2008; Zhang *et al.*, 2012). DCL2, DCL3, and DCL4 catalyse the cleavage of exogenous RNAs with double-stranded features into 22-, 24- and 21-nt siRNAs, respectively (Dunoyer *et al.*, 2005; Donaire *et al.*, 2008; Axtell, 2013).

We found that the silencing efficiency of both *PDS* and *CHS* reporters affected by silencing or overexpression of *PhERF2* was associated with substantial changes in abundance of silencing-associated genes, including *RDR2*, *RDR6*, *DCL2*, and *AGO2*. It appears likely that *PhERF2* modifies the transcription of these genes by binding *cis*-elements in their promoters. This possibility requires further examination in the future.

Many researchers have examined the effects of modulating expression of genes in the silencing complex on disease resistance. In *Nicotiana benthamiana*, for example, RDR6 was shown to be critical in resistance to CMV (Mourrain *et al.*, 2000) and *Potato virus X* (PVX) (Schwach *et al.*, 2005). In Arabidopsis, *DCL* mutants accumulate higher titers of TCV and CMV (Bouché *et al.*, 2006), TRV (Donaire *et al.*, 2008), *Turnip mosaic virus* (TuMV) (Garcia-Ruiz *et al.*, 2010), and *Tobacco mosaic virus* (TMV) (Lewsey and Carr, 2009). Our observations of increased and reduced TRV accumulations in *PhERF2*-silenced (Fig. 4E) and -overexpressing (Fig. 5E) petunias, respectively, and of the negative correlation between *PhERF2* expression and susceptibility to CMV (Fig. 6) are consistent with these findings, and support the hypothesis that *PhERF2* plays a regulatory role in the antiviral RNA silencing process. Mutants of *RAP2.2*, the Arabidopsis homolog of *PhERF2*, showed increased susceptibility to *Botrytis cinerea*, while its overexpression resulted in enhanced resistance to this pathogen (Zhao *et al.*, 2012). It seems possible that *PhERF2* might be involved in a general response to disease pressure, and future studies will examine the effect of up- and down-regulation of this transcription factor on susceptibility to fungal pathogens.

We were particularly interested in the roles of *PhERF2* and ethylene in plant growth and development and stress responses. Expression analysis demonstrated that *PhERF2* was also up-regulated in plants treated with the hormones SA, ABA, ethylene, and MeJA. These regulators sometimes function as growth inhibitors, and the interplay between *PhERF2* and these regulators may explain the substantial reduction in growth rate that was observed in silenced petunias (Fig. 4A) or the improvement of plant growth in the overexpressing lines (Fig. 5A). Although petunia corollas are ethylene sensitive (Reid and Wu, 1992), there was no significant change in flower longevity in transgenic plants where *PhERF2* was silenced or overexpressed (see Supplementary Table S2). Presumably *PhERF2* is not involved in ethylene-triggered floral senescence. We did, however, find that abiotic factors, such as cold, salt, and drought, enhanced the levels of *PhERF2* (Fig. 1C). This is consistent with studies on *PhERF2* homologs in other species. *NtCEF1* from tobacco is induced by cold and salt (Lee *et al.*, 2005). Salt treatment increases mRNA levels of *JERF1* (Zhang *et al.*, 2004) and *JERF3* (Wang *et al.*, 2004a) in tobacco, and plants overexpressing these genes were more tolerant of salt stress. *CaPF1* in pepper is rapidly and strongly activated under cold and salt conditions, and upregulation of this gene confers freezing tolerance (Yi *et al.*, 2004). Future work will include a study of the response of silenced and overexpressing plants to a range of abiotic stresses.

Our results suggest that *PhERF2* plays an important role in antiviral RNA silencing. The identification of PhERF2 as a positive regulator in the TRV-based VIGS system may provide a valuable solution to enhance the VIGS response when silencing efficiency is low, as has been observed in some species in which the TRV-based VIGS system has been tested (e.g. woody species) (Shen *et al.*, 2015). Thus it may be possible to transiently overexpress the PhERF2 or homologous proteins simultaneously or prior to the inoculation of silencing vectors. Or the inoculated plants could also be treated with ethylene or SA to increase the endogenous *ERF2*-like expression, which may lead to enhancement of the silencing efficiency. It is also worth mentioning that enhanced *PhERF2* expression by low temperature may lead to improvement of gene silencing efficiency in VIGS systems. This notion is supported by the fact that gene silencing efficiency is enhanced by low temperature with a TRV-based VIGS system in tomato (Fu *et al.*, 2006) and with a geminivirus-mediated VIGS system in cotton plants (Tuttle *et al.*, 2008). It would also be interesting in the future to examine whether PhERF2 plays any positive roles in other VIGS systems.

## Supplementary data

Supplementary data are available at *JXB* online.

Figure S1. Complete sequence of petunia *PhERF2* cDNA.

Figure S2. Amino acid sequence analysis of petunia PhERF2.

Figure S3. A proposed model for the roles of PhERF2 in antiviral RNA silencing.

Table S1. Primers used for semi-quantitative and real-time quantitative RT-PCR.

Table S2. The longevity of attached flowers from WT, *PhERF2*-RNAi and -overexpressing plants.

Supplementary Data
