# Supplementary material for: A petunia ethylene-responsive element binding factor, PhERF2, plays an important role in antiviral RNA silencing
Source: J Exp Bot. 2016 Apr 19;67(11):3353–65. doi: 10.1093/jxb/erw155 (PMC4892726; doi:10.1093/jxb/erw155)
Supplement: Supplementary Data [file supp_67_11_3353__index.html]

A petunia ethylene-responsive element binding factor, PhERF2, plays an important role in antiviral RNA silencing — A petunia ethylene-responsive element binding factor, PhERF2, plays an important role in antiviral RNA silencing — Supplementary Data 

# A petunia ethylene-responsive element binding factor, *PhERF2*, plays an important role in antiviral RNA silencing

## Supplementary Data

Data files

- supplementary\_figures\_S1\_S3\_tables\_S1\_S2.pdf - Supplementary Data
