# Supplementary material for: A petunia ethylene-responsive element binding factor, PhERF2, plays an important role in antiviral RNA silencing
Source: J Exp Bot. 2016 Apr 19;67(11):3353–65. doi: 10.1093/jxb/erw155 (PMC4892726; doi:10.1093/jxb/erw155)
Supplement: Supplementary Data [file supp_erw155_supplementary_figures_S1_S3_tables_S1_S2.pdf]

gctttttcatcaatcttttccatcaaaaatttac
tctatcaaaaagcccaatctttactttaaaaaaaaaaattgagctgagtgaatataaatc
*atg*tgt**ggtggtgctataatttccgattacatacctccgaaccggacttctcgccggtta**
M C G G A I I S D Y I P P N R T S R R L
accgctgagttactatggggtcgttccgatctgagtaaaaaatcaaaaaatccaagtaat
T A E L L W G R S D L S K K S K N P S N
tatcattcaaagcctttgagatctcaaatagttgaccttgacgatgacttcgaggctgat
Y H S K P L R S Q I V D L D D D F E A D
tttcaagagtttaaagatttttcagatgatgaagatgttaaaccatttgctttttctgct
F Q E F K D F S D D E D V K P F A F S A
cccaaacagtccactggctccaaatctgtaaaatctgctgattcagagaaggatgctgat
P K Q S T G S K S V K S A D S E K D A D
agttcctctaagagaaagaggaagaatcagtataggggggatcagacagcgaccttggggt
S S S K R K R K **N Q Y R G I R Q R P W G**
aagtgggcagctgaaatacgtgacccaagtaaaggtgttcgagtctggcttggaaccttc
K W A A E I R D P S K G V R V W L G T F
aatactgcagaagaagctgccagagcttatgatgttgaggctagaaggatcagaggcaat
N T A E E A A R A Y D V E A R R I R G N
aaagctaaggtgaatttccctgatgaagcgccagtgccatcttcaagacgcgctgttaag
K A K V N F P D E A P V P S S R R A V K
gtgaatcctcagaaggtccttcctagcctggactctgttcagccagacactactgtgatg
V N P Q K V L P S L D S V Q P D T T V M
aac**aacctgaggaatggctattatgattctttgggatttcttgaagagaaacctgtggca**
N N L R N G Y Y D S L G F L E E K P V A
aagcagtatggatatgaggatgggggcagtacttctgtagatataggattcgactcattt
K Q Y G Y E D G G S T S V D I G F D S F
gcgccttcagctggtgctgatatttacttcaactccgatgtgggaagcaactctttcgac
A P S A G A D I Y F N S D V G S N S F D
tgctctgactttggttggggagagccgtgcaccaggactccagagatatcatctgttctt
C S D F G W G E P C T R T P E I S S V L
tcagctgctatagaaagtaatgaagctcaatttgttgaagatgcccgtccagagaaaaaa
S A A I E S N E A Q F V E D A R P E K K
ctgaaatcagaccccaacaatccagtagctgatgatggaaacactgtgaacgagctatct
L K S D P N N P V A D D G N T V N E L S
gaagagctttcagcatttgaatctgagatgaagtttcttcagataccttatctggagggg
E E L S A F E S E M K F L Q I P Y L E G
aattgggatgcatcagctgataccttcctcaacacaagtgcaactcaggatggtgaaaat
N W D A S A D T F L N T S A T Q D G E N
gctatggacctctggtcatttgatgatgttccttctttgatgggaggtgtctac*taa*gtc
A M D L W S F D D V P S L M G G V Y −
agagtgccttccttgcctagcttttgtaaataaggcgtcatgtctgtctggtgatcgaaa
gaagttgatccac

Fig. S1. Complete sequence of petunia *PhERF2* cDNA. The cDNA sequence of *PhERF2* possesses an open reading frame (ORF) of 1,137-bp nucleotides encoding a protein of 378 amino acids. The 291-bp fragment used for VIGS construct was marked with solid underline while the 339-bp fragment (fragment 2) used for VIGS confirmation and RNAi construct was marked with dashed underline. The conserved AP2/ERF domain from amino acids 109 to 167 was indicated with bold types shaded in grey.

A

```
PhERF2     MCGGAIISDYIPPNRTSRRLTAELLWGRSDLSKKSKNPSNYHS-KPLRSQIVDLDDDFEADFQEFKDFSDDE------------DVKPFAFSAPKQS--TGSKSV-   90AA
PhERF3     MCGGAIISDLVPPSRTSRRLTADLLWGSSDLKK---NPSNYHSKPLRSSKFIDLDDEFEADFQDFS----------------------------DDIKPFGSKSV-   74AA
NtCEF1     MCGGAIISDYIAPSRTSRRLTAELLWGRSDLSNKQKNPNNYHS-KPLRSQVVDLDDDFEADFQDFKDFSDDEDVQ--------VDVKPFAFSASKNSNVEGSKSV-   96AA
SlJERF1    MCGGAIISDLVPPSRISRRLTADFLWGTSDLNKKKKNPSNYHSKPLR-SKFIDLEDEFEADFQHFKDNSDDDD---------------------D-VKAFGPKSV-   82AA
CaPF1      MCGGAIISDLVPPSRISRRLTAELLWGNSDLSKKKKNPGNYYSKPLNRSKFIDLDEEFEADFQDFKDYADDDV---------------------DDVKPFGSKSV-   84AA
StRAP2.12  MCGGSIISDYIDPNRTSRRLTAELLWGRFDLSKKQKNPNNYHS-KHLR---SEVVDDFEADFQDFKELSDDEDVE--------VDVKPFAFSASKQS--TGSKSS-   91AA
PsERF2a    MCGGAIISDFIAP-TRSR-LTADYLWP-----DLKKPSSGKRFSKPLRPEIVGLGDDFEADFQEFKDESDVDEDDEM------VEAKPSAFSAGKPSSARGSTTV-   92AA
MdRAP2.12  MCGGAIISDFIAP-VRSRRLTADYLWP-----DLKKPSSGKRLSKPLKSEIVDLDDDFEADFQEFKDESDVGEDDEM------VDSKPSAFSAGNPSFARGSTAV-   93AA
NnRAP2.12  MCGGAIISDFIPT-SRTRRVTADYLWP-----DLKNTTSGDYYSKPRRSEIDDLEEDFEADFQEFKDESEEEEED-----------IKPILLNSKPGLSRGSTTV-   88AA
AtRAP2.12  MCGGAIISDFIPP-PRSRRVTSEFIWP-----DLKKNLKGSKKSSKNRSNFFDFDAEFEADFQGFKDDSSIDCDDDFDVGDVFADVKPFVFTSTPKPAVSAAAEG-   99AA
AtRAP2.2   MCGGAIISDFIPP-PRSLRVTNEFIWP-----DLKNKVKASKKRSNKRSDFFDLDDDFEADFQGFKDDSAFDCEDDD---DVFVNVKPFVFTATTKPVASAFVSTG   97AA
           ****:**** : .    :  :* : :*       .            .  :****** *.                                .

                                              AP2/ERF domain
PhERF2     ---------KSADSEKDAD-SSSKRKRKNQYRGIRQRPWGKWAAEIRDPSKGVRVWLGTFNTAEEAARAYDVEARRIRGNKAKVNFPDE----APVPSSRRAVKVN   182AA
PhERF3     ---------KSADANCEEADKSSKRKRRNQYRGIRQRPWGKWAAEIRDPRKGIRVWLGTFNSAEEAARAYDVEARRIRGNKAKVNFPDE----TPVSASRRTVKQN   167AA
NtCEF1     ---------KTDDSDKDAD-RSSKRKRKNQYRGIRQRPWGKWAAEIRDPRKGVRVWLGTFNTAEEAARAYDVEARRIRGNKAKVNFPDE----APVPASRRTVKVN   188AA
SlJERF1    ---------RSGDSNCEAD-RSSKRKRKNQYRGIRQRPWGKWAAEIRDPRKGIRVWLGTFNSAEEAARAYDAEARRIRGKKAKVNFPDE----APVSVSRRAIKQN   174AA
CaPF1      ---------KSGDSSCDTE-KSSKRKRKNQYRGIRQRPWGKWAAEIRDPRKGIRVWLGTFNSAEEAARAYDVEARRIRGKKAKVNFPDG----SPASASRRAVKPN   176AA
StRAP2.12  ---------KTVDSDKDAADKSSKRKRKNQYRGIRQRPWGKWAAEIRDPRKGVRVWLGTFNTAEEAARAYDIEARKIRGKKAKVNFPDE----APAPASRHTVKVN   184AA
PsERF2a    ---------KSVEFN-GQAEKSAKRKRKNQYRGIRQRPWGKWAAEIRDPRKGVRVWLGTFNTAEEAARAYDSEARRIRGKKAKVNFPDE----TPRSSAKRSVKAS   184AA
MdRAP2.12  ---------KSVEFD-GQAEKSAKRKRKNQYRGIRQRPWGKWAAEIRDPRKGVRVWLGTFNTAEEAARAYDAEARRIRGKKAKVNFPEE----TPCASAKRSIKEN   185AA
NnRAP2.12  ---------KSVEVS-GPAEKSAKRKRKNQYRGIRQRPWGKWAAEIRDPRKGVRVWLGTFNTAEEAARAYDAEARRIRGKKAKVNFPEE----APQTAQKHTSKPN   180AA
AtRAP2.12  -----SVFGKKVTGLDGDAEKSANRKRKNQYRGIRQRPWGKWAAEIRDPREGARIWLGTFKTAEEAARAYDAAARRIRGSKAKVNFPEE---NMKANSQKRSVKAN   197AA
AtRAP2.2   IYLVGSAYAKKTVESAEQAEKSSKRKRKNQYRGIRQRPWGKWAAEIRDPRKGSREWLGTFDTAEEAARAYDAAARRIRGTKAKVNFPEEKNPSVVSQKRPSAKTNN   203AA
                   :.          *::***:******************** :* * *****.:*********  **:***.*******:              : . .

PhERF2     PQKVLP--SLDS--VQPDTT----VMNNLRNGYYDSLGFLEEKPVAKQYGYEDGGSTSVDIGFDSFAPSAGAD-IYFNSDVGSNSFDCSDFGWGEPCTRTPEISSV   279AA
PhERF3     PQ--KVEESSNT--VQLNTN----FCNNLDSGCYDSLDFVEEKPTAKQYAYEDVCITAGDMGLGSFSPSAGAD-VYFTSDEGSNTFDCSDFGWGEPGSRTPEISSV   264AA
NtCEF1     PQKVLPKEILDS--VQPDST----IINNMEDCCYDSLGFLEEKPMTKQFGCEDGSSASGDTGFGSFAPSAGTD-IYFNSDVGSNSFDCSDFGWGEPCARTPEISSV   287AA
SlJERF1    PQKALREETLNT--VQPNMT----YISNLDGGSDDSFSFFEEKPATKQYGFENVSFTAVDMGLGSVSPSAGTN-VYFSSDEASNTFDCSDFGWAEPCARTPEISSV   273AA
CaPF1      PQEALREEILNT--VQPNTT----YINNLDGGSDDSFGFFEEKPAAKQYGYENVSFTAGDMGLGSISPSTGTTNVYFSSDEGSNTFDCSDFGWGEPCPRTPEISSV   276AA
StRAP2.12  PQKVLPEESLDS--LQSDSA----IMN---SDHYDSFGFFEEKPMTKQYGYENGSSASADTGFGSFVPSVGGD-IYFNSDVGSNSFECSDFGWGEPCSRTPEISSV   280AA
PsERF2a    -QKMLPKTNMNA--IQPNLNQNINFVHDPNQDYYNAMGFLDEKPATNDFGFMSTFPANGEVVLKSSAPSDAVP-LYFSSDQGSNSFDCSDFGWGEQGSKTPEISSV   286AA
MdRAP2.12  PQKLIAKTNLNG--TQSNPNQNFNFVNDSSEDYYSALGFLDEKPTMNNFRYMSTFPANEDVALKSSVPSENAP-FYFSSDQGSNSFDCSDFGWGEQGSKTPEISSV   288AA
NnRAP2.12  PQNVLSKPNS------VSEKHSFNFTNNPDANFYDTMGFVEEKTSIKGSGYMSSFPVIG-------NPAEFKP-FAFNSDQESNSFSCSDFSWGDQEPKTPEISSV   272AA
AtRAP2.12  LQKPVAKPNP------NPSPALVQNSNISFEN----MCFMEEKHQVSNNNNNQFGMTNS------VDAG-CNGYQYFSSDQGSNSFDCSEFGWSDQAPITPDIS--   284AA
AtRAP2.2   LQKSVAKPNKSVTLVQQPTHLSQQYCNNSFDNSFGDMSFMEEKPQMYNN---QFGLTNS------FDAGGNNGYQYFSSDQGSNSFDCSEFGWSDHGPKTPEIS--   298AA
            *                                 : *.:**        .    .            ..        *.**   **:*.**:*.*.:   . **:**

PhERF2     LSAAIESNEAQFVEDAR-PEKKLKSDPNNPVADDGNTVNELSEELSAFESEMKFLQIPYLEGNWDASAD--TFLNTSA---TQDGENAMDLWSFDDVPSLMGGVY-   378AA
PhERF3     LSAVAEGNGTQFDEDAS-PAKRLKSCTNNSVQDDGNTVDKLSEELSAFESQMKFLQIPYLEGNWEASVD--TLLNADA---TQEVGNAMDLWSFDDVPSLMGGVY-   363AA
NtCEF1     LSAVIESNESQLVEDDTSPMKKLKSSPINPVADDGNTANKLSEELSAFETQMKFLQIPYLEGNWDASVD--TFLNSSA---TQDGDNAMDLWSFDDVPSLLGGVF-   387AA
SlJERF1    LSEVLETNETHFDDDSR-PEKKLKSCSSTSLTVDGNTVNTLSEELSAFESQMKFLQIPYLEGNWDASVD--AFLNTSA---IQDGGNAMDLWSFDDVPSLMGGAY-   372AA
CaPF1      LSEVLECNGTQSDEDAR-PEKKLKSCSNASLPDEDNTVHTLSEELSAFESQMKFLQIPYLEGNWDASVD--AFVNTGA---IQDGGNAMDLWPSMMFLL-------   369AA
StRAP2.12  LSAAVDSNEAQFVEDAN-PQKKLKSCTNNPVADDGNTVNMLSEELPAFAPQMNFFHLQYMEGNWDASVG--NFLNTSA---TQNGENAMDLWSFDDVPSLMGGIF-   379AA
PsERF2a    LSSVMEESDDSLFLEDANPTKKLKSNPEDLVPVQDNAGKTLSDELSAFE--MKYFQTPYLGGSWDASVD--AFLSADA---TQDGGNSVDFWSFDDLIGGGF----   381AA
MdRAP2.12  ISSVMEESDNSPFLEDSNPTKKMKPNSQDLEPPEDNSGKTLSDELSAFE--MKYFQTPYLDESWDASVD--AFLNGDA---TQDGGNPMDLWSFDDLPAIVGGVF-   386AA
NnRAP2.12  LSTAIE-SDGSEFLEDANPKKKLRTGSEGVAPIEESTPNTLSEELSAFESQMKFLQMPYLEGSWDASID--SLFSGEP---TQDAENSIDLWSFDDLVPPTVGGSY   372AA
AtRAP2.12  -SAVINNNNSALFFEEANPAKKLKSMDFETP--------------------------YNNTEWDASLD---FLNEDAVTTQDNGANPMDLWSIDEIHSMIGGVF-   358AA
AtRAP2.2   -SMLVNNNE-ASFVEETNAAKKLKPNSDESDD---------------------LMAYLDNALWDTPLEVEAMLGADAGAVTQEEENPVELWSLDEINFMLEGDF-   379AA
            *   :  .       :   .*:::.                                    *::.       :..   .      ::  *.:::*.    .
```

B

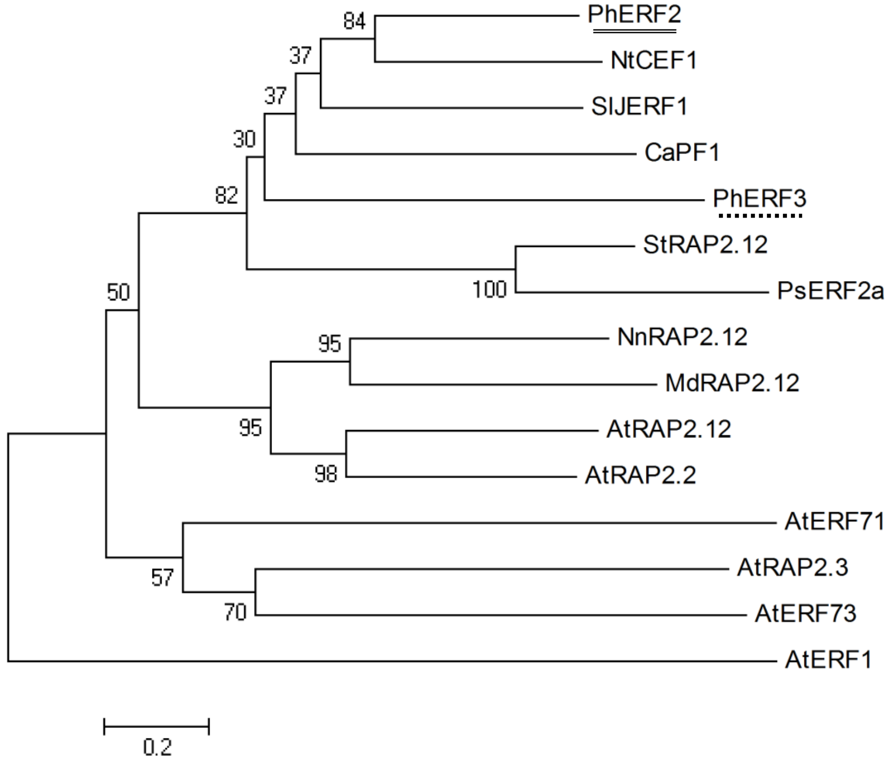

Fig. S2. Amino acid sequence analysis of petunia PhERF2. (A) Alignment of deduced PhERF2 amino acid sequence and other similar proteins from *Petunia × hybrida* PhERF3 (ADP37418), *Nicotiana tabacum* NtCEF1 (AAP40022), *Solanum lycopersicum* SlJERF1 (NP_001234513), *Capsicum annuum* CaPF1 (AAP72289), *Solanum tuberosum* StRAP2.12 (XP_006342909), *Prunus salicina* PsERF2a (ACM49847), *Nelumbo nucifera* NnRAP2.12 (XP_010273830), *Malus domestica* MdRAP2.12 (NP_001280975), *Arabidopsis* AtRAP2.12 (NP_175794) and AtRAP2.2 (NP_566482). Identical amino acids are shown with asterisks. AP2/ERF domain is indicated in square. (B) Phylogenetic tree of petunia PhERF2 aligned with *Arabidopsis* group VII ERFs and homologs from other plant species. Phylogenetic relationship of PhERF2 with *Arabidopsis* AtRAP2.12 (NP_175794), AtRAP2.2 (NP_566482), AtERF71 (NP_182274), AtRAP2.3 (NP_188299), AtERF73 (NP_001077812), *Petunia × hybrida* PhERF3 (ADP37418), *Solanum lycopersicum* SlJERF1 (NP_001234513), *Capsicum annuum* CaPF1 (AAP72289), *Nicotiana tabacum* NtCEF1 (AAP40022), *Solanum tuberosum* StRAP2.12 (XP_006342909), *Prunus salicina* PsERF2a (ACM49847), *Nelumbo nucifera* NnRAP2.12 (XP_010273830), *Malus domestica* MdRAP2.12 (NP_001280975). PhERF2 is highlighted with doubled underlines. PhERF3, one paralog of PhERF2 in petunia, was used for VIGS control in this study and marked with a dashed line. The tree was constructed using the neighbor-joining method with MEGA software (version 4.0.2). *Arabidopsis* AtERF1 (NP_567530), one member of group IX ERFs, was used as the out-group. Boot-strap values were generated with 1000 replicates and shown at corresponding branch nodes.

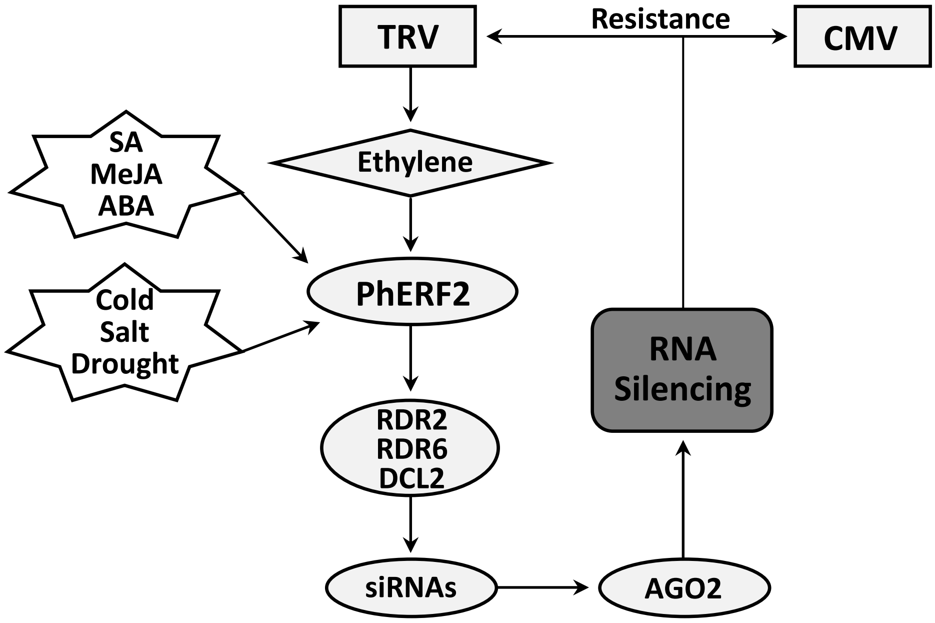

Fig. S3. A proposed model for the roles of PhERF2 in antiviral RNA silencing. Expression of *PhERF2* is induced by multiple factors, including ethylene, SA, MeJA, ABA, cold, high salinity and water deficit. PhERF2 positively influences RDR2, RDR6, DCL2, AGO2 expressions to improve siRNAs accumulation and enhance RNA silencing, which may trigger a broad resistance to TRV and CMV infections.

Table S1. Primers used for semi-quantitative and real-time quantitative RT-PCR.

Primer name	Accession no.	5'—3'	Product size
vigsPhERF2(F1)	HQ259596	GGTGGTGCTATAATTTCCGATTA	291bp
vigsPhERF2(R1)		AGCATCCTTCTCTGAATCAGCAG	
vigsPhERF2(F2)	HQ259596	AACCTGAGGAATGGCTATTATGA	339bp
vigsPhERF2(R2)		GTTTCCATCATCAGCTACTGGAT	
vigsPhERF3(F1)	HQ259597	GAATTTGAAGCTGACTTTCAGGA	246bp
vigsPhERF3(R1)		ATAAGCTCTGGCAGCTTCTTCTG	
PDS(F1)	AY593974	CAGTGCTTCTTGATCGCTTTGAA	138bp
PDS(R1)		TCTGACTTGGCCACCTTTTGACT	
PDS(F2)	AY593974	CGAATAAGAAAGATCGAGCTGAA	130bp
PDS(R2)		CAGGCAAAAGAAGCTTGAAAATA	
CHS(F1)	X14599	ACCATTGGGCATTTCTGACTGGA	197bp
CHS(R1)		ATGAAGCCTTTCTCATTTCATCC	
CHS(F2)	X14599	CTTGGATAGTTTGGTTGGACAAG	202bp
CHS(R2)		AGGAACATCTTTGAGCAAGTGAA	
TRV1(F1)	AF406990	CAGTCTATACACAGAAACAGA	463bp
TRV1(R1)		GACGTGTGTACTCAAGGGTT	
TRV2(F1)	AF406991	GGTTACTAGCGGCACTGAATAGA	225bp
TRV2(R1)		TAGTACTCCCTTGGTTCGTCGTA	
TRV2(F2)	AF406991	ACGGACGAGTGGACTTAGATTC	Depending on the inserted fragment
TRV2(R2)		GTTTAATGTCTTCGGGACATGC	
RDR1(F1)	CV299561	TCATGATGTCACAATTGAGGAAG	271bp
RDR1(R1)		TTCGGAGTGATAGGTTGTCTTGT	
RDR2(F1)	FN008007	ACGATATGGTGGTTACAAAGGTG	190bp
RDR2(R1)		ATCCTCCACTCCCAAAGTAGAAA	
RDR6(F1)	GT007757	TTTGCTGATCGGGAACTATTAAA	277bp
RDR6(R1)		CATATGACTTTCTTGCCACTTCC	
DCL1(F1)	FN028672	GGAGAAAGTTGAACAGGATGATG	412bp
DCL1(R1)		CAACATCGACATCCAATACAATG	
DCL2(F1)	FN020290	TGGAACCTCTGACATCGTAGAAT	322bp
DCL2(R1)		ATATTCTGGAGGAAGAAGCCATC	
DCL3(F1)	GBDQ01037961	GGAGACTTGTAAATTCTACGATC	206bp

DCL3(R1)		TTTCAAGGCAACACAATATCTTT	
DCL4(F1)	FN002500	AGGATATGATGAAACACCCTGTG	222bp
DCL4(R1)		TAGCCTTTGAACCTGCATTAGAG	
AGO1(F1)	CV298592	TCTATCAAAGTATCCGCCCTACA	246bp
AGO1(R1)		CTTGAGATGTTAAACCCGAGATG	
AGO2(F1)	FN018838	GTTCATCGGAGCTGATGTTAATC	371bp
AGO2(R1)		GAAATAGCCGTGTATGGTGTCTC	
AGO4(F1)	FN031884	GGAGGGTCCTAGATAGAGTGCAT	368bp
AGO4(R1)		ATGCTGCCTCAAGATTATATCCA	
rnaiPhERF2(F1)	HQ259596	ATACTAGTGGCGCGCCAACCTGAGGAA TGGCTATTA	371bp
rnaiPhERF2(R1)		ATGGATCCATTTAAATGTTTCCATCATCA GCTACTG	
oePhERF2(F1)	HQ259596	ATCTCGAGATGTGTGGTGGTGCTATAAT	1159bp
oePhERF2(R1)		ATGAGCTCTTAGTAGACACCTCCCATCA	
CMVcp(F1)	EU315691	ACTGGTTTATCAGTACGCTGCAT	150bp
CMVcp(R1)		ACTAACTCGTCTGTCTCGAGTGC	
26S rRNA(F1)	AF479174	AGCTCGTTTGATTCTGATTTCCAG	185bp
26S rRNA(R1)		GATAGGAAGAGCCGACATCGAAGG	

Table S2. The longevity of attached flowers from WT, *PhERF2*-RNAi and -overexpressing (OE) lines.

	Flower longevity (days ±SD)
Wild-type	6.94 ±0.58
PhERF2-RNAi (#1)	6.88 ±0.65
PhERF2-RNAi (#4)	6.81 ±0.56
PhERF2-OE (#C)	7.06 ±0.46
PhERF2-OE (#D)	6.94 ±0.68
PhERF2-OE (#I)	7.00 ±0.43

Ten flowers from three individual plants for each line were used for studying flower longevity. The time from anthesis to completed wilting of the corolla was recorded. No differences among all the lines tested were observed.
